# The role of behavioral nudges in sustaining public health engagement through the “Tawakkalna” app: insights from healthcare professionals

**DOI:** 10.3389/fpubh.2026.1803030

**Published:** 2026-07-07

**Authors:** Fahad Al-Anezi

**Affiliations:** Department Management Information Systems, Imam Abdulrahman Bin Faisal University, Dammam, Saudi Arabia

**Keywords:** behavior, digital health, engagement, mHealth, nudges, Saudi Arabia, sustainability

## Abstract

**Background:**

Digital health applications, especially those deployed by the governments have become essential tools for managing public health. Several such applications were launched during the COVID-19 pandemic as a part of crisis management, such as Tawakkalna in Saudi Arabia, some of which are continued operating in the post-pandemic period with the introduction of additional services. While behavioral nudges such as reminders, alerts, and notifications in such applications have demonstrated effectiveness in improving health behaviors in previous studies, they are mostly focused on adoption and usability aspects and limited to pandemic period, rather than long-term engagement.

**Purpose:**

This study aims to investigate how behavioral design features, such as reminders, alerts, and tailored health notifications, embedded in the Tawakkalna application influence sustained public health engagement in the post COVID-19 pandemic period.

**Methods:**

A qualitative exploratory design using semi-structured interview technique was adopted in this study. A total of 24 participants from diverse roles including healthcare professionals, digital health strategists, and policymakers participated in the interviews. Braun and Clarke's thematic analysis framework was used to identify key patterns and themes related to behavioral design, engagement, and governance considerations.

**Results:**

Six themes emerged from the analysis of interview data which included personalization and adaptive nudging, engagement dynamics, ethical and trust considerations, system integration, social and community influences, and future directions. Results reflected that adaptive personalization countered notification fatigue; engagement varied by age, context, and emotional framing; ethical concerns emphasized transparency and cultural alignment; system integration enhanced credibility; social/family influences amplified impact; and future designs advocated AI-driven, bidirectional nudges within preventive health journeys.

**Conclusion:**

To achieve sustained engagement, applications like Tawakkalna should integrate behavioral nudges which are adaptive, transparent, and socio-culturally attuned.

## 1 Introduction

The rapid developments in the digital health technologies, especially in the area of artificial intelligence (AI) have significantly transformed the healthcare systems across the globe. These developments have facilitated improved disease surveillance, health communication, and public engagement. The COVID-19 pandemic has led to the immediate need of launching digital health applications, as a result, many countries have deployed digital health tools like mHealth applications to address public health challenges (1, 2). Tawakkalna, is one such digital health application developed by Saudi Data and Artificial Intelligence Authority (SDAIA) during the COVID-19 pandemic, for facilitating real-time infection tracking, vaccination services, mobility permits, and official healthcare communication (3). The application wide-scale adoption during the pandemic made it as a trusted digital interface between the citizens and public health system.

However, as the pandemic subsided, the applications functionalities and features were expanded in order to shift the focus from emergency use to sustained use. Accordingly, the application now offers more than 1,000 government and public related services, which can be accessed by both citizens and residents (4). The services offered were expanded across healthcare, education, judiciary, tourism, and transportation (5, 6). These developments are facilitated by digital health transformation process as a part of Saudi Arabia's Vision 2030 initiative, which aims to transform various sectors through digitalization, using innovative technologies (7). The application has been further improved with the integration of AI to improve the efficiency and effectiveness of the services offered, making it a leading example of AI-driven digital governance and long-term public service innovation (8).

Extensive research has been conducted on the application's effectiveness and efficiency in different contexts. Behavioral nudges integrated in digital health applications have reflected a significant improvement in sustained health engagements and preventive behaviors (9–17). Nudges such as reminders, prompts, notifications, feedback etc. are guided by persuasive design principles, providing guidance toward the desired behaviors without coercion (9). For instance, behavioral nudges have shown significant improvements in vaccination (10), medication adherence (11), chronic-disease self-management (12), and sustained engagement with digital health applications (13, 14). Furthermore, personalization feature in applications can enhance the effectiveness of nudges by increasing relevance of messages, thereby reducing notification fatigue (15, 16). However, it is to be understood that sustained engagement is also influenced by other factors such as perceived usefulness, perceived ease of use, trust etc., apart from the nudges (17). Behavior change technique taxonomies have identified feedback and monitoring, goal-setting and planning, associative strategies, and personalization as important components of interventions that support adherence. Techniques such as prompts, goal-setting are overrepresented as effective interventions; whereas behavioral aspects such as self-monitoring reflected weaker impact on adherence outcomes. It has been observed that most intervention in the previous research, combined multiple behavioral change techniques, without effectively considering the nudge components such as timing, frequency, tailored or personalized reminders, which also account for sustained engagement (18, 19).

In the context of Saudi Arabia, digital health applications like Tawakkalna were mostly investigated for their role during the COVID-19 pandemic. Tawakkalna application was found to be effective in areas like contact tracing, permit management, and reduced infection rates (20–22). In addition, perceived usefulness, ease of use, and trust were identified to the significant influencers of adoption (23–25). In addition, its effectiveness was realized in improved access to testing, vaccination, and telehealth services (26); but it was associated with challenges such as data privacy, security, decline in engagement after the pandemic (27). However, the previous studies considered nudges as operational or compliance factors; and rarely theorized them as structured behavioral nudges. In addition, emerging features such as tailored or personalized health reminders, feedbacks, tele-health links, and organ-donation prompts were not effectively investigated for their influence on health and preventive behaviors, after the pandemic subsided (28–30).

Recent regional and international research has shown that sustained engagement with digital health applications depends not only on access to technology, but also on app design, user expectations, usability, trust, and personalization. An experimental study in 2021 (31) proved that high-prototypicality application design improved perceived usefulness, ease of use, and usage intentions, indicating that design attributes influence adoption beyond one's content. Similarly, findings from mHealth engagement study (32, 33) suggested that users' expectations on navigation, settings and feature placement are significantly associated with attention and propensities to use health applications. A detailed analysis of engagement with smartphone applications implied that enhanced health outcomes were linked to increased app engagement, although retention was variable according to context. Recent research (2022–2026) has highlighted that engagement is multifactorial, proposing that successful digital health solutions must incorporate reminders, feedback, personalization, and user-centered design, as well as address attrition, contextual needs, and socioenvironmental inequities (34–37). Overall, these studies support the argument that nudges on national platforms like Tawakkalna are more likely to keep users engaged if they are adaptive, trustworthy, and culturally aligned, rather than generic or static.

Existing studies concentrate on adoption intentions, usage during the pandemic or digital government performance. What is missing is how the specific features of nudges, like reminders, alerts and personalized health notifications, are designed, perceived and leveraged to sustain long-term public health participation in Saudi Arabia. Moreover, while behavioral nudges increase engagement in digital health contexts, their impact in national integrated platforms such as Tawakkalna has not been investigated. Additionally, stakeholder perspectives, especially those from policymakers, healthcare providers, and digital-health strategists, are seldom explored, constraining the knowledge on design and governance factors for sustained engagement. Capturing behavioral nudges in Tawakkalna fills critical gaps in the post-pandemic digital health literature, with implications for national platforms globally.

Therefore, the present study aims to investigate the effect of behavioral design features (i.e., reminders, alerts, and tailored health notifications) embedded in the Tawakkalna application on the sustained public health engagement beyond the COVID-19 pandemic in Saudi Arabia. The paper is structured as follows. In Section 2, behavioral features vs. nudges and the role of Tawakkalna in public health engagement is discussed. Section 3 outlines the qualitative methods (participant profiles, interview protocol, and Braun & Clarke analysis). Section 4 provides six emergent themes. Section 5 discusses findings and theoretical/practical implications. Section 6 concludes with recommendations.

## 2 Theoretical and contextual foundations

### 2.1 Conceptualizing behavioral features and digital nudges in mHealth

In this study, it is important to distinguish between broader behavioral features of mHealth applications and more targeted digital nudges embedded within them. Behavioral features refer to any app functionalities that can shape health-related behavior, such as appointment booking, access to tele-consultations, health assessment tools, or dashboards that visualize risk and service use over time (38, 39). These features alter the choice environment by making certain health actions more visible, convenient, or efficient, but they do not necessarily prompt users at a specific moment or in a specific manner. Digital nudges, by contrast, are intentionally designed prompts or cues—such as reminders, alerts, notifications, and tailored messages—that steer users' attention or decisions toward desirable health behaviors while preserving freedom of choice (40). In Tawakkalna, examples include vaccination reminders, medication alerts, organ-donation prompts, outbreak warnings, and targeted health campaign messages that are timed and framed to encourage preventive actions rather than merely informing users about available services. While behavioral features create the infrastructure for engagement (for example, enabling tele-health visits or access to medical reports), nudges operate on top of this infrastructure to activate, sustain, or redirect user engagement at key moments in the care journey (41).

Conceptually, this distinction is important for understanding public health engagement in practice. Behavioral features support structural access to health services at scale, whereas digital nudges can foster repeated use, adherence to recommendations, and participation in population-level interventions (such as vaccination campaigns, screening programmes, or outbreak control measures) (42). In the context of a national platform like Tawakkalna, which has evolved from a crisis-response tool to a multi-service digital ecosystem, the interplay between features and nudges is expected to shape how citizens continue to interact with public health services beyond the pandemic. This study therefore focuses on how specific nudge mechanisms—particularly reminders, alerts, and tailored health notifications—are perceived, designed, and governed by key stakeholders, and how these mechanisms can support a transition from emergency-driven usage to sustained, preventive public health engagement.

From a developmental perspective, early versions of Tawakkalna primarily relied on mandatory or compliance-oriented behavioral features (for example, movement permits or status checks) to ensure adherence to COVID-19 regulations. As the application has expanded to include various services, the role of digital nudges has shifted toward encouraging voluntary, preventive behaviors in domains such as vaccination, chronic disease management, and organ donation. By examining how experts imagine the future design of these nudges, this study provides cues on how Tawakkalna and similar platforms may evolve into long-term engines of public health engagement rather than short-term crisis tools.

### 2.2 Public health engagement through digital health applications: the case of Tawakkalna

Public health engagement refers to the sustained participation of individuals and communities in preventive health behaviors, service utilization, and population-level interventions, such as vaccination uptake, adherence to chronic disease management protocols, participation in screening programmes, and response to outbreak alerts (43). Digital health applications support this process through behavioral design features (including nudges) by reducing friction in access to services, providing timely prompts to act, and creating feedback loops that reinforce positive behaviors. For example, reminders and alerts can bridge intention-behavior gaps by prompting users at optimal moments (e.g., before a vaccination deadline), while tailored notifications leverage personalization to increase relevance and reduce fatigue, ultimately fostering habitual engagement with public health systems.

In Tawakkalna—a national platform developed by the Saudi Data and Artificial Intelligence Authority (SDAIA)—these mechanisms are embedded across over various services in healthcare. Key behavioral features and nudges include medication reminders, vaccination appointment alerts, organ donation prompts, tele-health booking notifications, health assessment result alerts, outbreak warnings, and crowd density visualizations for pilgrims (44). The app also integrates family-linked notifications (e.g., household vaccination status) and personalized health campaigns based on user profiles. Currently used by over 34 million citizens and residents, with 1 million daily active users across 77 countries, Tawakkalna's healthcare services—such as ambulance requests, medical report access, and preventive screening reminders—are particularly sought by working-age adults (25–55 years) for routine care, families for vaccination and child health services, and pilgrims for travel-related health checks. Usage data show high engagement in health campaigns (e.g., seasonal flu vaccination) with most interactions being driven by push messages rather than passive browsing (44–48).

Table 1 shows an overview of healthcare service usage patterns of Tawakkalna based on recent studies (49–51). The table maps these high-use domains to current behavioral features (e.g., embedded records, self-monitoring dashboards) and nudge enhancement opportunities (e.g., family alerts for vaccination, personalized insights for health status). This profile outlines key areas for nudge optimization to maintain post-pandemic public health engagement: COVID vaccination follow-through, chronic care adherence, and pro-social behaviors such as organ donation.

**Table 1:** Tawakkalna usage profile.

Health service	Tawakkalna functionalities	Usage %	Behavioral features	Nudge opportunities	Engagement priority
COVID vaccine	Status checks, bookings	87.2%	Records integration	Reminders, family alerts	High (preventive)
Health services	Telehealth, assessments	53%	Virtual consult access	Post-assessment prompts	High
Health test gate	Pre-travel screening	42.6%	Risk visualization	Outbreak warnings	Medium
Health status	Personal health dashboard	29.8%	Self-monitoring tools	Personalized insights	Medium
Public donation	Organ donation registration	41.8%	Pledge forms	Values-based prompts	High (pro-social)

Performance is monitored through SDAIA dashboards tracking metrics like daily active users, notification open rates (typically 60%−80% for health alerts), service completion rates (e.g., 90% for vaccination bookings post-reminder), and user feedback scores (4.5/5 on app stores) (52). While the app performs strongly in compliance-driven contexts (e.g., 95% permit check compliance during COVID), post-pandemic engagement has shifted toward voluntary preventive use, with challenges in sustaining interest for non-urgent services (53). This evolution highlights the potential of refined nudge strategies to support long-term public health engagement, which this study examines through expert perspectives.

## 3 Methods

### 3.1 Study settings and participants

Saudi Arabia faces a dual burden of health challenges, transitioning from infectious diseases to a predominance of non-communicable diseases (NCDs) such as diabetes (prevalence ~18.3%), obesity (~35%), cardiovascular disease, and hypertension, driven by rapid urbanization, dietary shifts, and an aging population (54). These issues necessitate scalable preventive strategies, including sustained public engagement in screening, medication adherence, vaccination, and lifestyle interventions—areas where digital health applications like Tawakkalna play a critical role by integrating nudges for behavior change within national platforms aligned with Vision 200′s digital health transformation goals. Development needs for such apps include adaptive reminders, personalized alerts, and integration with primary care to address low adherence rates (e.g., < 50% for chronic medications) and support population level preventive health journeys.

The study adopted a qualitative exploratory design to explore the influence of behavioral nudges embedded in the Tawakkalna application on sustained public health engagement. This approach is suitable to explore the subtle, context-dependent perceptions of behavioral and motivational factors by experts, with rich insights into design preferences, ethical considerations, and implementation challenges that cannot be fully captured by quantitative metrics (55–57). The success of the Tawakkalna nudge is supported by quantitative evidence, for example 87% vaccination appointment completion post reminder (49) but the questions—^*^why/how^*^ these work in Saudi Arabia's sociocultural contexts of family collectivism, religious health framing, urban/rural divides and post-COVID notification anxiety. Accordingly, participants included healthcare professionals, public health officials, digital health strategists, and experts involved in the design of Tawakkalna or similar applications. These participants were selected because they are well-equipped with knowledge of digital health governance, behavioral design features, and population-level engagement during and after the COVID-19 pandemic.

### 3.2 Recruitment and sampling

A purposive sampling strategy was adopted to ensure the inclusion of participants with diverse professional roles and relevant expertise in digital health, public health, and health policy. The initial target sample size was informed by the study's aim and by a prior estimate of 21 participants as observed in similar studies (58). However, consistent with qualitative research principles, recruitment continued until thematic saturation was reached, meaning that no new concepts or insights were emerging from the interviews. Thematic saturation was reached after interview 24, when the final three interviews generated no new codes or themes. Saturation was operationalized as the absence of new substantive codes during iterative comparison of each transcript with the existing coding framework.

### 3.3 Data collection

Semi-structured interviews were conducted through secure online video conferencing (average duration: 40–50 min). Interviews were guided by the core questions shown in Table 2 below, each designed to elicit expert insights on specific dimensions of behavioral nudges while allowing flexibility for emergent discussion. The interviews were conducted in both Arabic and English according to participant preference. Seven interviews were conducted in Arabic and 17 in English. The Arabic interviews were transcribed verbatim and translated into English by a professional translator for analysis and reporting. The researcher reviewed the translated excerpts against the original Arabic transcripts to preserve meaning consistency and resolve any ambiguous wording before finalizing the quotations used in the manuscript. The interviews were audio-recorded with participant consent.

**Table 2:** Core interview questions and rationales.

Serial number	Core question	Rationale
1	“What specific behavioral nudges (e.g., reminders, alerts, notifications) have you observed in Tawakkalna, and how do users typically respond to them?”	To capture observed nudge types and real-world engagement patterns from expert experience
2	“How do personalization, timing, and emotional framing influence the effectiveness of these nudges for sustained public health engagement?”	To explore design factors (personalization, context) affecting long-term behavior change
3	“What ethical challenges (e.g., privacy, autonomy, manipulation) arise from nudge implementation, and how can they be addressed?”	To identify governance considerations critical for trust and scalability
4	“How does Tawakkalna's integration with other systems (e.g., healthcare, family accounts) enhance or hinder nudge impact?”	To assess technical and ecosystem factors influencing nudge delivery
5	“What future nudge features or strategies would you recommend to sustain engagement beyond crisis-driven usage?”	To generate forward-looking recommendations grounded in stakeholder expertise

### 3.4 Data analysis

Thematic analysis was conducted following Braun and Clarke's (59) six-phase framework, applied iteratively using NVivo V.14 for systematic data organization, coding, and theme development. Phase 1 (Familiarization) involved multiple readings of transcripts by two researchers, I.A. and T.A., to immerse in data meanings. In phase 2, (initial coding), 187 initial codes were inductively generated from patterns in participants' responses (e.g., “notification fatigue,” “family decision-making”). In phase 3, theme development, codes were grouped into 18 sub-themes (e.g. adaptive timing, ethical transparency) and the sub-themes were further refined into 6 main themes through team discussion. In Phase 4 (Theme review) themes were compared back to the entire data set and participant quotes to ensure coherence. Phase 5 (Theme definition) Theme names and nudge effectiveness narratives finalized. The results of Phase 6 (Reporting) are shown in Supplementary Table A1 (see Appendix A). This analysis was conducted using NVivo using query matrices (co-occurrence of codes), word clouds for salience and framework matrices that mapped themes onto the domains of expertise of the participants. The domains of participants systematically affected the development of themes: Theme 3 (Ethical considerations) was primarily influenced by policy/ethics experts (68% codes from policy/IT domains), Theme 2 (Engagement dynamics) was driven by clinical practitioners (72% from healthcare roles), and Themes 1 & 6 (Personalization/Future) were predominantly contributed to by digital strategists (65% from tech domains). NVivo framework matrices mapped domain-theme contributions, with Theme 3 showing highest multidisciplinary sensitivity (input across all domains).

### 3.5 Ethical considerations

This study adhered to strict ethical guidelines including voluntary participation, confidentiality, and informed consent. Ethical approval (IRB-2025-14-0852) was obtained from institutional review board Imam Abdulrahman Bin Faisal University before conducting the interviews. Participants were fully informed about the interview's procedure, their rights before starting the interviews.

#### 3.5.1 Reflexivity and positionality

The study was conducted by a researcher affiliated with a Saudi institution with experience in health research, providing contextual understanding of the national digital health landscape and requiring continuous reflexive awareness throughout the research process. This professional background allows for rapport with the participants and assists in the interpretation of institutional and policy related issues, but may have influenced the expectations of the platform and its use. The researcher was not formally affiliated with Tawakkalna, SDAIA, or any of the related governance bodies and was not involved in the design or administration of the platform. Reflexive practices were employed throughout the study, including a semi-structured interview guide, documenting analytic decisions, and repeated comparison of emerging themes with original transcripts in order to reduce interpretive bias and maintain fidelity to the participants' perspectives.

## 4 Results

A total of 24 interviews were conducted with participants from diverse fields including public health, clinical practice, digital health strategy, health informatics, policy, cybersecurity, user experience, and community engagement (see Supplementary Table A2, Appendix A). Participants included 11 males and 13 females. Age of the participants ranged from 29 to 52 years. Professional experience ranged from 5 to 26 years.

Thematic analysis of interview data resulted in six key themes (see Table 3), which are discussed below. Each theme systematically addresses the core interview questions: Theme 1 (Personalization) directly responds to Q1 (observed nudges) and Q2 (design factors); Theme 2 (Engagement) elaborates Q2 effectiveness mechanisms; Theme 3 (Ethics) comprehensively covers Q3 governance challenges; Theme 4 (Integration) answers Q4 system factors; Theme 5 (Social) addresses contextual influences from Q2/Q3; and Theme 6 (Future) provides Q5 recommendations. This alignment demonstrates comprehensive coverage of the study protocol.

**Table 3:** Emergence of themes.

Theme	Sub-themes	Codes	Example quotes (from transcripts)
**1. Personalization and adaptive behavioral nudging**	•Adaptive nudging •Personalized content •Context-sensitive nudges •Family-centered personalization	•Dynamic timing •Predictive nudges •Emotional framing •Cultural framing •Household decision-making •Real-time analytics	“The system should predict when users need a service rather than reminding them after they miss it.” (P2) “Patients respond better when nudges emphasize well-being rather than procedures.” (P3) “One notification can trigger a collective decision within families.” (P13)
**2. Engagement dynamics and user response patterns**	•Motivational preferences •User fatigue/saturation •Behavioral differences (age, region) •Emotional/psychological impact	•Gamification •Positive reinforcement •Notification fatigue •Timing sensitivity •Anxiety triggers	“Generic reminders quickly lead to ‘notification blindness.”' (P13) “Young adults respond only when nudges align with exam or travel seasons.” (P10) “Red notifications still trigger anxiety because people associate them with COVID.” (P18)
**3. Ethical, trust, and governance considerations**	•Transparency & autonomy •Ethical risks •Trust-building mechanisms •Cultural/ethical alignment	•Consent renewal •Behavioral profiling •Nudge explanation •Data reassurance •Religious framing	“Users should see why they received a message to avoid feeling manipulated.” (P11) “Adaptive nudging can unintentionally become hidden personalization.” (P11) “Organ donation nudges require ethical framing to be accepted.” (P23)
**4. System integration and technical factors**	•Data quality & architecture •Nudge orchestration •Cross-channel coordination •Multi-sector integration	•System responsiveness •Avoiding nudge collision •Clinical alignment •Voice-enabled nudges	“Nudges are only as strong as the data feeding them—outdated records break trust.” (P4) “We created a nudge-orchestration layer to prevent overload.” (P15) “Nurses now ask about Tawakkalna notifications during triage.” (P20)
**5. Social and community-level influences**	•Community-oriented nudging •Social mediation & family negotiation •Religious/cultural reinforcement •Professional mediation	•Social norms •Collective responsibility •Intergenerational decision-making •Counseling triggers in pharmacies	“People respond to cues about how their community is performing.” (P1) “Notifications become negotiation tools within families.” (P22) “Health nudges resonate more when echoed in mosque sermons.” (P23)
**6. Future directions for advanced behavioral design**	•Predictive & AI-driven nudging •Integrated preventive health journeys •Bidirectional digital interaction •National behavioral ecosystem	•AI micro-nudges •Life-course health pathways •User-initiated nudging •Policy-linked nudges	“We're testing AI models that reduce frequency when users show fatigue.” (P24) “We need to shift from isolated nudges to long-term health journeys.” (P16) “Users should be able to nudge the system back.” (P10)

### 4.1 Personalization and adaptive behavioral nudging

Experts identified personalization as the key factor, with interviewees reporting that sustained engagement depends on adaptive nudges. Many participants observed that static reminders are no longer relevant in the post-pandemic context, and argued that nudges should respond to real-time user patterns, geographic context, and personal health needs.

Furthermore, several participants stressed the importance of emotional framing, reflecting their view that nudges should not be instructional, but be encouraging. For instance, a senior physician stated that “patients respond better when nudges emphasize wellbeing rather than procedures“. Also, digital strategists highlighted the need for predictive nudging for analyzing the users' needs before their disengagement. Accordingly, one of them stated that the system should predict when users need a service rather than reminding them after they miss it”.

Moreover, family-centered personalization was also highlighted by few participants, reflecting their opinion that households in Saudi Arabia commonly make collective health decisions. Stressing the importance of behavioral nudge, one of the participants stated that “one notification can trigger a collective decision within the family”.

Further perspectives indicated that users are more likely to respond positively when nudges are presented during active use of the application. One health informatics specialist said, “Behavior-triggered nudging is more effective because the message appears when the user is already engaged with the platform”. Similarly, another participant noted that “nudges should follow the rhythm of the user rather than the schedule of the system”, highlighting the importance of timing and behavioral context.

Participants also commented on the tone of notifications. One patient experience specialist said “messages that recognize stress or difficulty feel supportive instead of demanding” and another expert noted that “human-like empathy cues make the platform seem caring rather than administrative”. These findings suggest that emotional sensitivity and supportive communication styles become increasingly important in sustaining long-term engagement.

Some participants also mentioned the intergenerational dynamics in families. “Older family members often depend on younger relatives to interpret notifications and act on them,” one regional healthcare administrator noted, suggesting that behavioral nudges may have an indirect effect on multiple users in a household setting.

### 4.2 Engagement dynamics and user response patterns

Significant differences were observed in relation to the engagement and user response patterns across the age groups, digital literacy levels, and contextual user needs. About two-thirds of the participants highlighted that the effectiveness of repetitive or generic notifications has been gradually declining, as a result of nudge fatigue or ignoring notification, resulting from overexposure. In addition, half of the participants stated that young users of the application tend to engage continuously, only if the nudges coincide with meaningful personal events such as travel, examinations, or seasonal health campaigns. For instance, a primary care director described that “young adults respond only when nudges align with exam or travel seasons“. Engagement was also perceived to be influenced by emotional responses. About fourteen participants observed that users' reaction are still influenced by the pandemic related associations. For instance, a digital health expert mentioned that red notifications still trigger anxiety because people associate them with the COVID-19 pandemic”, and recommended softer visual cues and supportive language. A shift toward motivational digital strategies was observed by the participants, as few participants referred gamification, progress indicators, positive reinforcement as effective strategies for long-term engagement.

Other participants mentioned that repetitive reminders lose their effect, as users start to mentally block them out. One participant called this “behavioral saturation from predictable messaging patterns”, and another explained that “generic reminders quickly become background noise”. Similarly, a public health specialist said that “people eventually stop noticing notifications if they pop up too often”.

Several interviewees also spoke about emotional avoidance behaviors related to health notifications. One participant said that “patients who experienced stress during the pandemic sometimes avoid opening health notifications altogether,” while another expert said that “behavioral residue from the pandemic continues influencing user perception of alerts”. These findings highlight that patterns of digital engagement are conditioned by previous experiences of pandemics.

The participants also noted that the degree of engagement differs substantially across various demographic groups. A pharmacist said that “older adults are more consistent, especially if the reminder is about medication or an appointment”, and a digital entrepreneur explained that “younger users ignore preventive messages unless they relate to something happening in their daily lives”.

### 4.3 Ethical, trust, and governance considerations

The majority of interviewees raised ethical concerns, emphasizing transparency and user autonomy as key aspect, several experts raised concerns about hidden personalization, terming it as ‘implicit consent drift' as users usually forget what they initially agreed to, and this may result in privacy issues without their knowledge. Participants who were specifically in the roles related to ethics, policy and digital security have raised concerns with respect to data misuse, behavioral profiling, and risk labeling. For instance, one of the participants warned that “adaptive nudging can unintentionally become hidden personalization”.

Few participants highlighted that regular communication about data protection and security initiatives would ensure that trust is maintained among the users. Almost half of the participants believed that if the users are explained ‘why they receive specific nudges', would reduce their perceptions about manipulation. In addition, religious and cultural framing was highlighted by few participants as they highlighted that nudges regarding organ donation, preventive screenings, or community health behaviors must align with local values to maintain legitimacy.

Other findings showed that transparency was strongly associated with user trust and continued engagement. “Users disengage when the purpose behind notifications is unclear,” stated another policy expert, while one participant argued that “people should be able to see exactly how their data informs the nudges they receive”. Responses indicate users expect a higher degree of explainability and transparency in digital health systems.

Ethical sensitivity also correlated with the tone and framing of nudges. A behavioral scientist stated that “nudging must preserve dignity and avoid creating pressure or guilt”, suggesting that too aggressive reminders can have a negative effect on the user's trust. Another participant commented that “some nudges require ethical and religious sensitivity so as not to provoke public resistance”, notably in the case of preventive screening and organ donation.

The need for consent to be renewed periodically was also mentioned by some participants. One cybersecurity expert noted that users may “forget over time what they agreed to initially,” adding that users may slowly lose awareness of what permissions they approved. This finding highlights the increasing relevance of ongoing ethical governance in digital health systems.

### 4.4 System integration and technical determinants of nudge effectiveness

Nudge credibility was one of the important factors perceived by the majority of the participants, which can influence trust. Technical issues such as data lags, or nudges with inaccurate data can lead to users' perception that they are irrelevant or incorrect, thereby reducing their trust. In this context, one of the participants stated that “Nudges are only as strong as the data feeding them—outdated records break trust“. The importance of cross-channel coordination involving physicians, pharmacists, and nurses was highlighted by moist of the participants, stressing that clinical acknowledgment of digital nudges would enhance compliance. Furthermore, multi-sector integration, such as using nudge-based incentives through insurance or linking with smart-home systems was highlighted by few participants.

Other participants mentioned that the responsiveness of the system directly affects how users interpret behavioral nudges. As one technology specialist explained, “technical responsiveness affects whether users see nudges as useful or intrusive”, implying that delays and technical inconsistencies undermine user confidence.

Cross-platform consistency was also identified as an important factor influencing engagement. “Patients get confused when clinical advice and app reminders don't match,” said one nurse informaticist. Another participant said that “patients respond better when nurses reinforce app notifications during consultations”. These findings indicate that the integration between healthcare professionals and digital systems increases user confidence and compliance.

Pharmacists and frontline health practitioners were also seen as key mediators in the interpretation of nudges. “many users ask pharmacists to interpret notifications they receive”, said one pharmacist, pointing to the ongoing importance of human support in the face of increasing digitisation. Another participant stressed the importance of real-time integration and accurate synchronization of health data, saying that “outdated records immediately erode confidence in the notifications.”

### 4.5 Social and community level influences

Socio-cultural factors were considered as a strong motivator for the participants. About two-thirds of the participants expressed that social norms, family decision-making, and community messaging can significantly improve users' responsiveness to nudges. For instance, one of the public health officials stated that “people respond to cues about how their community is performing”, thereby recommending that community-level dashboards could significantly improve the engagement in long-term. Furthermore, cultural and religious influence was noted by very few participants. One of the participants who collaborates with religious institutions noted that “health nudges resonate more when echoed in mosque sermons“, indicating the influence of religion on health behaviors.

Additional findings revealed that collective social identity plays an important role in digital health engagement in Saudi Arabia. “Before anything happens, notifications tend to be discussion points within households,” said one participant, suggesting that health decisions are often socially shared and negotiated.

Other interviewees also spoke about community-driven reinforcement. “People are more likely to stick to preventive behaviors if they think other people around them are also doing so,” a behavioral expert said. Another participant said, “Community-level engagement creates a sense of collective responsibility rather than individual obligation”.

Some participants also discussed the role of trusted intermediaries in reinforcing nudges. One healthcare professional said “health messages are more persuasive when delivered by community leaders or health workers” and another participant said “social trust increases the effectiveness of digital reminders”.

### 4.6 Future directions for advanced behavioral design

Majority of the participants highlighted that nudging should be integrated with AI models. Specifically, predictive, micro-personalized or customized nudges which adjust tone, frequency, and modality based on user emotion and behavior were considered to be key aspects that need to be integrated in the near future. For instance, one such application as noted by a data scientist was that they were testing an AI model that reduce frequency when users show fatigue, so that users are not irritated. Participants also suggested bidirectional nudging, with users able to request guidance or initiate actions, reflecting the integration of generative AI models such as ChatGPT, for real-time and effective communication. In addition, most of them stressed the need to embed nudges within long-term preventive health journeys, but not just isolated reminders.

Other participants said that future behavioral systems need to be more proactive and predictive. “One digital strategist said, ‘AI needs to identify decline in engagement earlier than the user goes all the way out'. Another said, ‘Future nudges need to continually adapt based on user behavior and emotional state'.”

The concept of conversational and interactive nudging was also stressed. One participant pointed out that “users should be able to communicate back to the system, instead of passively receiving reminders,” highlighting the growing expectations for interactive digital health experiences. Another expert similarly said, “AI-driven conversational support could make health engagement feel more natural and less administrative.”

Moreover, many participants anticipated the possibility of implementing behavioral nudges within larger preventative health care ecosystems. One policy advisor explained, “nudges should become part of lifelong preventive health pathways rather than isolated interventions”. Another participant noted, “future digital health systems should combine behavioral science, AI and community engagement into one integrated framework”.

## 5 Discussion

The findings in this study extended the existing evidence on digital nudging from experts point of views, reflecting that the digital health tools such as Tawakkalna, which were launched during the COVID-19 pandemic for managing public health, need to evolve from static prompts toward adaptive, emotionally sensitive, and context-aware engagement mechanisms in the post-pandemic phase. This finding can be correlated with international evidence highlighting the effectiveness of personalized nudges in enhancing digital health adherence (15, 16). Previous studies have evaluated such nudges (feedback, monitoring, and goal-setting) in controlled intervention contexts (9–14), but the findings in this study extend this understanding by explaining how personalization must be integrated in a complex national platform, used extensively for healthcare and other government-related services on a daily-basis. Strong preferences for adaptive and context-specific nudges, by focusing on timing, emotional framing, and alignment with users' life contexts are now critically important to address issues such as notification fatigue and “blindness” to generic reminders, and to enhance long-term engagement. This shift in perceptions supports previous findings that perceived usefulness and trust are important predictors of long-term engagement (17); however, these factors alone are insufficient and must be complemented by adaptive, personalized, and emotionally resonant nudges to sustain engagement. One of the key findings which is less visible in prior studies which have primarily focused on individual acceptance factors such as perceived usefulness, ease of use, and trust (20, 23–25), was adaptive and family-centered personalization for achieving sustained engagement, especially in a collectivist context where household decision-making is common. This finding suggests that national platforms in tightly knit social structures should not only rely on individualized behavioral techniques but also consider shared norms, and religious reinforcement to manage long-term engagement.

Experts reported observing that user responsiveness varied by age, emotions, and context, with interviewees emphasizing timing, frequency, and message framing as key determinants of behavioral effectiveness (18, 19). Participants specifically noted emotional fatigue, pandemic trauma, and notification overload as significant barriers to sustained engagement. One of the interviewees stated that: ‘Red notifications still trigger anxiety because people associate them with COVID, reflecting how experts perceived pandemic-era visual cues continue influencing user response patterns. This finding highlights that historical emotional associations were perceived to influence present engagement patterns.

Although ethics, transparency, and governance were observed as the factors relating to privacy and data security, limiting the users' engagement and adoption in previous studies (23–25), the participants in this study identified more nuanced risks such as “implicit consent drift,” hidden personalization, and anxiety triggered by crisis-associated visual cues (for example, red alerts), arguing for “explainable” nudging where users understand why they receive specific prompts. This highlights the need for accountable and transparent behavioral design along with a context-specific layer in which religious and cultural framing are seen as important factors to be considered in the design process.

The study while emphasizing the prior Tawakkalna related research, reflecting the application's effectiveness in contact tracing, permit management, and service access (20–22, 26), also highlights that technical factors like data quality, cross-channel coordination, and AI-driven orchestration shape the credibility of behavioral prompts were not effectively analyzed in previous studies. Participants' suggestions for predictive, AI-driven micro-nudges, life-course health journeys, and bidirectional interaction reflect a transition from perceiving Tawakkalna as a static application to envisioning it as a national behavioral ecosystem—an advancement not previously reflected in earlier, pandemic-centric assessments (13, 14, 20, 28–30). Priority developments for Tawakkalna informed by study findings and Saudi public health needs include: AI-driven personalization, Family/religious framing, Ethical transparency dashboards, Cross-sector orchestration. These align with Vision 2030 priorities for NCD prevention (diabetes/obesity) and sustained engagement beyond crisis response.

### 5.1 Study limitations

This study has several limitations that must be taken into account when interpreting the results. First, the study relied on experts' views rather than lay users of Tawakkalna which means that the findings are based on knowledgeable professional interpretations of behavioral nudges, but may not fully reflect the real users' lived experiences, expectations, and challenges. Secondly, the study employed a qualitative exploratory design with purposive sampling and thus the findings are context specific and not meant for statistical generalization. Third, although the participants had diverse professional backgrounds, the sample may not be equally representative of all relevant stakeholder groups, particularly frontline users, rural populations and those with limited digital literacy. Fourth, although the study offers a solid conceptual basis for understanding sustainable engagement with Tawakkalna, future research should include a second-phase study with end users and, where possible, comparative or mixed-method designs to validate and extend such findings. Finally, member checking was not conducted, so the themes were not returned to participants for confirmation. In addition, social desirability bias may have influenced responses, particularly because some participants were involved in the design or governance of the Tawakkalna platform.

### 5.2 Theoretical and practical implications

This study adds to the existing literature on behavioral design and digital health by demonstrating that successful engagement with national health apps is not only dependent on the presence of nudges but also on their personalization, contextual relevance, emotional relevance and culture fit. The findings contribute to the behavioral nudge theory by highlighting the importance of adaptive, predictive, and family-oriented nudging in collectivist settings, demonstrating that the effectiveness of nudging is contingent upon cultural integration and systemic trust rather than individual choice. The study further advances the technology acceptance models by demonstrating the influence of trust, openness to ethics and credibility of data on user responses to nudges as perceived by participants. Findings suggest that Tawakkalna and similar applications' developers should provide adaptive nudges based on the user's history and context, minimize redundant notifications, and include clear communication on the use of data. Policy makers and system architects should ensure that nudging tactics are compatible with cultural and religious norms, while at the same time allowing for cross-sector coordination and AI-driven personalization.

## 6 Conclusion

This study explored expert perceptions of behavioral nudges embedded in Tawakkalna and found that participants perceived personalization, trust, social context and system integration as important for maintaining public health engagement. The findings suggest that nudges may be more effective when they are adaptive, culturally relevant, and transparent in their delivery. However, these are interpretations based on expert perspectives, not lay users. So, future research should include a second phase study with end-users to understand how these design features are experienced in practice and to validate the present findings.

## Data Availability

The original contributions presented in the study are included in the article/Supplementary material, further inquiries can be directed to the corresponding author.
