# Supplementary material for: The role of behavioral nudges in sustaining public health engagement through the “Tawakkalna” app: insights from healthcare professionals
Source: Front Public Health. 2026 Jul 7;14:1803030. doi: 10.3389/fpubh.2026.1803030 (PMC13385168; doi:10.3389/fpubh.2026.1803030)
Supplement: Supplementary file 1 [file Supplementary_File_1.docx]

# Appendix A

Table A1: Application of Braun & Clarke Framework in NVivo V.14

| **Phase** | **Description** | **NVivo V.14 Application** | **Output** |
| --- | --- | --- | --- |
| 1. Familiarization | Repeated transcript reading | Import 24 transcripts; word frequency queries | 12,400 coded references identified |
| 2. Initial coding | Inductive code generation | Open coding → 187 nodes created | Codebook with definitions/examples |
| 3. Theme development | Code clustering | Codebook hierarchy; matrix coding query | 18 sub-themes → 6 main themes |
| 4. Theme review | Dataset validation | Framework matrix; constant comparison | Theme coherence confirmed (92% code-theme fit) |
| 5. Theme definition | Theme refinement | Mind maps; theme comparison | Final theme narratives + quotes |
| 6. Reporting | Extract selection | Export coded transcripts; word trees | Results section |

Table A2. Interviewees demographics

| **Participant** | **Role / Position** | **Age** | **Gender** | **Experience** | **Domain of Expertise** |
| --- | --- | --- | --- | --- | --- |
| 1 | Senior Public Health Official | 47 | Male | 22 years | Public health policy & epidemiology |
| 2 | Digital Health Strategist (National Digital Transformation Program) | 38 | Female | 12 years | Digital transformation & mHealth strategy |
| 3 | Consultant Family Physician | 44 | Female | 18 years | Primary care & patient behavior |
| 4 | Health Informatics Specialist (MOH) | 36 | Male | 10 years | Health informatics & data systems |
| 5 | Behavioral Scientist (National Center for Health Promotion) | 41 | Female | 14 years | Behavioral science & health promotion |
| 6 | Senior Epidemiologist | 50 | Male | 25 years | Epidemiology & outbreak management |
| 7 | CTO, Digital Health Startup | 45 | Male | 20 years | Digital health technology & startups |
| 8 | Senior Health Policy Advisor (CHI) | 52 | Female | 26 years | Health policy & governance |
| 9 | Lead UX Researcher (SDAIA) | 33 | Male | 9 years | User experience (UX) & app design |
| 10 | Deputy Director, Primary Healthcare Centers | 48 | Female | 23 years | Primary healthcare management |
| 11 | Cybersecurity & Digital Ethics Consultant | 40 | Male | 15 years | Cybersecurity & digital ethics |
| 12 | Director of Patient Experience, Tertiary Hospital | 37 | Female | 11 years | Patient experience & service design |
| 13 | Senior Public Health Official | 46 | Female | 20 years | Public health administration |
| 14 | Consultant Cardiologist & Digital Lead | 39 | Male | 13 years clinical; 4 years digital | Clinical cardiology & digital health |
| 15 | Digital Health Architect (National Platform Team) | 34 | Male | 10 years | Digital platform architecture |
| 16 | Behavioral Scientist (Academic–Government Advisor) | 41 | Female | 15 years | Behavioral science & policy advising |
| 17 | Regional Primary Care Director | 52 | Male | 26 years | Primary care leadership |
| 18 | Data Protection & Ethics Officer | 37 | Female | 9 years | Data protection & ethics |
| 19 | Community Pharmacist | 33 | Female | 10 years | Community pharmacy & health counselling |
| 20 | Nurse Informaticist | 36 | Male | 12 years nursing; 5 IT | Nursing informatics & clinical IT |
| 21 | HealthTech Entrepreneur (Tawakkalna Partner) | 42 | Male | 15 years | HealthTech entrepreneurship & partnerships |
| 22 | Family Physician (Semi-rural Region) | 45 | Female | 18 years | Rural primary care & digital adoption |
| 23 | Imam Collaborating on Health Campaigns | 50 | Male | 25 years | Community health & religious outreach |
| 24 | Digital Health Data Scientist | 29 | Female | 5 years | AI/ML & behavioral analytics |
